# Pandemic Education—Insights into Teachers’ Perceptions of Hygiene Measures in Schools Due to COVID-19

**DOI:** 10.3390/ijerph20065207

**Published:** 2023-03-16

**Authors:** Flora Woltran, Katharina-Theresa Lindner, Susanne Schwab

**Affiliations:** 1 Centre for Teacher Education, University of Vienna, Porzellangasse 4, 1090 Vienna, Austria; 2 Department of Education, University of Vienna, Sensengasse 3A, 1090 Vienna, Austria; 3 Optentia Research Focus Area, North-West University, 1174 Hendrick Van Eck Boulevard, Vanderbijlpark 1900, South Africa

**Keywords:** masked education, COVID-19, facemasks, hygiene measures

## Abstract

To prevent the spread of Coronavirus (COVID-19) and protect the health of school staff and students, Austrian education policymakers introduced several hygiene measures that posed new challenges for teachers. The current paper focuses on teachers’ perceptions of hygiene measures in schools during the 2021–2022 school year. In Study 1, 1372 Austrian teachers participated in an online survey at the end of 2021. In Study 2, five teachers participated in an in-depth qualitative interview study. The quantitative results show that half the teachers felt a strong burden from the COVID-19 tests, but that the tests worked better when teachers had more teaching experience. Elementary and secondary school teachers, unlike special education teachers, had fewer problems implementing COVID-19 testing. The qualitative results suggest that teachers needed an acclimatization period for previously unfamiliar tasks, such as COVID-19 testing, to become accustomed to this newly implemented measure. Additionally, wearing facemasks was only evaluated positively in the context of self-serving strategies, while the protection of student health was not considered. In summary, the current study calls attention to the particular vulnerability of teachers and provides insights into the reality of schools in times of crisis that could be particularly helpful to education policymakers.

## 1. Introduction

### 1.1. Austria’s Education Policy Measures during the COVID-19 Pandemic

Due to the coronavirus disease (2019) (COVID-19) pandemic that emerged in early 2020, school systems around the world plunged into an unprecedented crisis. In a total of 194 countries, either partial or full school closures were used as a measure to control the incidence of infection in educational institutions [1].

At the onset of the COVID-19 pandemic, Austria, like many other countries, responded quickly to the rapid spread of the new virus. From March until May 2020, a nationwide closure of schools went into effect. After several weeks of distance education, teachers and students were able to return to school in May 2020. However, vulnerable students were allowed to continue attending classes remotely. In addition, strict hygiene measures, such as wearing facemasks or washing and disinfecting hands regularly, were implemented throughout the return-to-school period.

With the start of the 2020–2021 school year, the Federal Ministry of Education, Science, and Research published a plan to comply with basic hygiene measures (e.g., disinfection of hands and regular ventilation of classrooms). However, stricter measures were reintroduced in schools in November 2020 due to a sharp rise in infection rates across the state. Accordingly, face-to-face school meetings and teacher conferences were prohibited from that time on. Further, students in ninth grade and above were sent to distance learning until the end of the month. Compulsory schools remained in face-to-face teaching. However, classes could transition to distance education if circumstances permitted (high numbers of COVID-19 infections). Schools remained open for mentoring and educational support as well as for vulnerable students, such as students with special educational needs or those attending German language support classes to improve their German language skills. After a period of opening and closing schools, students and teachers were required to participate in SARS-CoV-2 antigen testing up to three times per week in February 2021. In addition, facemasks had to be worn outside the classroom in elementary schools and also during lessons in lower secondary schools. In upper secondary schools, wearing FFP-2 facemasks was mandatory.

For the 2021–2022 school year, the Federal Ministry of Education, Science, and Research envisioned continuous in-person instruction without interruptions [2]. During the first three weeks of the beginning semester (September 2021), teachers as well as students of all ages and school types had to undergo SARS-CoV-2 antigen testing three times a week [3]. Depending on the state-specific COVID-19 incidence rate, either level 1 (no or low risk), level 2 (medium risk), or level 3 (high or very high risk) came into effect. Regardless of the current risk level, unvaccinated students and teachers or those not fully recovered from infection had to undergo SARS-CoV-2 antigen and Polymerase Chain Reaction (PCR) testing three times a week [3,4]. At risk level 2, students as well as teachers in elementary and lower secondary schools needed to wear surgical or FFP2 facemasks outside the classroom. In the event of exceptionally high infection rates (level 3), students in grade nine and above as well as their teachers were now required to wear FFP2 facemasks both inside and outside the classroom [3,4]. If a particularly high number of new infections affected individual classes or schools, the responsible educational board could order a transition to distance learning for certain classes or schools [3]. Due to the sharp increase in new COVID-19 infections in mid-November, new measures came into force for the school sector [4]. Accordingly, the Federal Ministry of Education published a decree stating that the decision as to whether a student attended in-person classes would be left to the student or their parents or guardians [4]. Additionally, elementary and lower secondary school students were required to wear surgical facemasks outside classrooms [4]. Students in ninth grade and above were required to wear FFP2 facemasks inside and outside the classroom [4]. Furthermore, all teachers, regardless of grade level, were required to wear FFP2 facemasks at all times [4]. After the Christmas holidays, students who wanted to attend classes in person were able to return to school in January 2022 [5]. Unvaccinated students and teachers still had to undergo weekly SARS-CoV-2 antigen and PCR testing [5]. Wearing facemasks outside the classroom was mandatory for all students and school staff [5]. Starting in February, the obligation to wear facemasks in classrooms was abolished for elementary school students. At the end of February, all students were required to attend class in person, except those infected with COVID-19 and those whose parents or guardians were in the at-risk group [6]. Finally, the requirement to wear facemasks was suspended in April [7]. Two months later, education policymakers decided to discontinue COVID-19 testing in schools [8].

### 1.2. Teachers’ Perceptions of Hygienic Measures in Schools due to COVID-19

Although the role of educational institutions in the SARS-CoV-2 infection process remains unclear [9], school closures were a last resort in many countries during the COVID-19 pandemic [10]. However, previous studies have shown that the suspension of in-person teaching has serious effects on the lives of learners (e.g., [10,11]). Accordingly, several findings point to a deterioration in children’s health and well-being and an increase in inequities between students from disadvantaged and more privileged households (e.g., [11,12,13,14]). In addition, previous evidence indicates that school closures put students at risk of educational and socioemotional losses [15]. To circumvent such drastic measures and associated negative outcomes, schools in many countries have implemented various measures, such as staggered schedules, switching between digital and face-to-face instruction, reduced class sizes, and hygiene measures [14]. In particular, the general use of facemasks, along with hand hygiene and social distancing, was found to be particularly effective in controlling infections [15].

Wearing facemasks is a comparatively easy measure to implement in schools [16] and is effective in containing the spread of the virus [17]. However, experts point out several potential physical and social disadvantages, especially for young children and people with respiratory diseases [15]. In addition, wearing facemasks can be challenging for students with special educational or healthcare needs (e.g., deaf or hard-of-hearing children) and their teachers [15]. In general, face masks can obscure a large portion of the face, interfere with verbal and nonverbal communication, and block emotional signals between teachers and students [18], which can negatively affect teacher–student relationships, group cohesion, and learning [17,19]. In addition, facemasks, when worn for prolonged periods, can cause and exacerbate headaches [20]. Because of this, and although wearing facemasks was mandatory in schools during COVID-19 in Austria, implementation in individual schools may have varied because of differences in the local epidemiologic situation and school and class characteristics. This, in turn, may have led teachers and learners to express doubts about appropriate behavior when using face masks [15]. With this in mind, school stakeholders’ views on the use of facemasks and perceptions of potential risks should be considered in light of future health crises [15].

Although several studies indicate various negative effects on social interactions between teachers and students associated with the wearing of face masks in the classroom [19,21], Spitzer (2021) [17] emphasizes that maintaining the health of all stakeholders should remain a top priority in schools. For example, Spitzer (2021) [17] suggests compensating for the loss of emotionality through facemasks by strengthening other forms of expression such as posture and gestures. In addition, Nobrega et al. (2020) [22] provide several recommendations for teachers to counteract potential communication problems, such as reducing ambient noise, speaking slowly and articulating correctly, or using features and visual aids as well as pictures in activities.

While wearing facemasks in schools poses several problems for student–teacher relationships, COVID-19 testing, considered a useful strategy for rapidly identifying and isolating infected individuals [11], is often fraught with bureaucratic obstacles. For example, in a study by Unger et al. (2021) [23], school principals indicated that school staff were not prepared for this new responsibility and did not have the necessary skills to administer the COVID-19 tests. Interestingly, Unger et al. (2021) [23] reported that teachers generally favor frequent COVID-19 testing. This is consistent with a study by Lorenc et al. (2021) [24], who found that the majority of school personnel believe that COVID-19 testing in schools is an important measure to ensure safety, but that it should take as little time as possible and be conducted in a non-disruptive manner. 

In general, regular COVID-19 testing can be an effective hygiene practice to prevent the uncontrolled and rapid spread of COVID-19 infections in schools. However, it also requires extensive preparation and planning, as well as additional time and space resources, and can therefore pose a logistical challenge for school administrators and teachers [25]. To reduce negative consequences for students and teachers (e.g., reduction in instructional time, bureaucratic burden), COVID-19 testing must be feasible and efficient [23]. In addition, in the event of a staff shortage at the schools, support staff must be deployed who have the necessary knowledge and skills to administer the COVID-19 tests.

### 1.3. Aim of the Current Study

Although previous studies (e.g., [19,20]) have highlighted several problems associated with the widespread implementation of hygiene measures (e.g., wearing of facemasks and regular COVID-19 testing), no studies are available that have examined differences in teachers’ perceptions depending on the type of school or looked at relationships between teaching experience, job satisfaction, and teachers’ satisfaction with the COVID-19 testing. Against this background, the current study explores teachers’ experiences with hygiene interventions during an ongoing educational crisis using the following four research questions:

1. How do teachers perceive the implementation of hygienic measures in their school?
2. Are there differences depending on the type of school the teacher teaches in (elementary school, secondary school, or special education school)?
3. Does teaching experience affect teachers’ satisfaction with the COVID-19 tests?
4. Is COVID-19 testing related to teacher job satisfaction?

## 2. Materials and Methods

### Project Description: Inclusive Home Learning Teachers’ Perspectives (INCL-LEA-T)

The current study’s data derive from a broader research project called Inclusive Home Learning Teachers’ Perspectives (INCL-LEA-T) [26], which can be divided into three measurement points: the first nationwide school lockdown in Austria (March to May 2020) (t1), the second nationwide school lockdown in Austria (November to December 2020) (t2), and new regulations ensuring “safe school operation” (November to December 2021) (t3). Some of the data used in the current study were used in a previously published paper by Lindner et al. [27]. However, the previously published work focused exclusively on trends in teachers’ job satisfaction and emotional adjustment performance over time, including other measurement time points. Items related to teachers’ perceptions of the COVID-19 measures were not used. Therefore, it does not provide information on teachers’ perceptions of the hygiene measures and how these perceptions relate to job satisfaction and subjectively perceived stress.

For the current paper, only data from t3 were included, as new infection control measures were implemented in Austrian schools at that time. The data collection included both a quantitative online questionnaire using LimeSurvey (Study 1) as well as guided interviews conducted via Zoom (Study 2). To capture the experiences of a large number of teachers, the links to the online survey as well as to the invitation to participate in the interviews were first sent to school principals in all Austrian states and were then forwarded to teachers.

## 3. Study 1

### 3.1. Participants

A total of 1372 teachers were recruited to participate in the online survey. However, only 1170 participants were included in the current paper, as they responded to the questions about COVID-19 testing. The remaining 202 participants did not give any answers to these questions. This is because skipping questions was optional when completing the questionnaire. Most participants were female (80.1% female, 19.5% male, 0.4% diverse), and the mean age was 45 years (M = 44.72; SD = 11.38), with a range of 21 to 64 years. This is broadly consistent with current population characteristics of the gender distribution among teachers in the Austrian education system. Accordingly, in the school year 2020/21 73.13% of the teachers were female [28]. The distribution by school type shows that 36.3% of the participating teachers teach in elementary schools, 27.5% in compulsory secondary schools (compulsory secondary schools are attended by students in grades 5–8; after graduation, students can transfer either to a secondary school or to a vocational preparatory school to complete the mandatory 9th grade or continue their school career until the 12th or 13th grade), 14.6% in academic secondary schools (academic secondary schools are attended by students in grades 5–12 and generally prepare students for postsecondary education), and 13.7% in special schools. The remaining 7.9% of teachers teach at other schools, such as vocational schools. This also roughly corresponds to the current population characteristics. According to Statistics Austria [28], 29% of teachers teach in elementary schools, followed by teachers in secondary compulsory schools (23.5%), teachers in upper academic secondary schools (18%), and teachers in special schools (4.23%) in the 2020/21 school year. In addition, participants had been working as teachers for an average of 18 years at the time of the study (Md = 18; M = 18.65; SD = 12.31), with a range of 0 to 45 years. The sample is not representative because teachers from elementary and special schools are over-represented, while teachers who teach at higher grade levels (both lower and upper secondary) are under-represented.

### 3.2. Procedure

The online survey included both closed- and open-ended questions about teachers’ perceived opportunities and challenges, as well as their general assessment of current working conditions in schools. To obtain information about teachers’ perceptions of the COVID-19 tests, the following three statements were given: “The COVID-19 testing process works well in my classroom”, “I feel a heavy burden from regular COVID-19 testing at my school”, and “My students feel a heavy burden from regular COVID-19 testing at school”. All of these statements had to be ranked on a four-point Likert scale ranging from 1 (strongly disagree) to 2 (partially disagree), 3 (partially agree), and 4 (strongly agree). Teachers’ job satisfaction was assessed using a four-point Likert scale developed by Enzmann and Kleiber (1989) [29]. All items were rated on a scale from 1 (strongly disagree) to 4 (strongly agree). The reliability for the current sample was Cronbach’s alpha = 0.82. Furthermore, exploratory factor analysis was performed (KMO = 0.86; Bartlett’s test = sig., *p* < 0.01). According to the scree plot and the eigenvalue criteria (eigenvalue > 1), one factor with an explained variance of 54.20% was found (λ = 0.59–0.82; communalities = 0.34–0.67).

### 3.3. Data Analysis

The quantitative data were analyzed using SPSS version 27 software. In addition to descriptive statistics, a Pearson correlation analysis was performed. The dependent variables deviated significantly from the normal distribution. However, due to the large sample size, a MANOVA was conducted to examine differences between school types. According to Finch (2005), the statistical method of multivariate analysis of variance is robust even if the requirement of a normal distribution of the data is violated [30]. The dependent variables were the three statements on the COVID-19 test, and the school type (elementary school, compulsory secondary school, academic secondary school, and special school) was used as the independent variable. A post-hoc Scheffé test was conducted to identify group differences. In interpreting effect sizes, we followed Cohen’s (1988) findings that Eta^2^ values around 0.01 indicate weak effects, Eta^2^ values around 0.06 indicate moderate effects, and Eta^2^ values around 0.14 indicate strong effects [31].

### 3.4. Results

Table 1 presents the descriptive statistics of teachers’ perceptions of the COVID-19 testing measures. Generally, the majority of teachers felt that the process worked well. Further, around half indicated that they felt high levels of burden due to the testing. For their students, teachers perceived lower levels of burden.

The results of the multivariate analysis of variance show a significant main effect for school type (F3, 1061 = 11.75, *p* < 0.01, Eta^2^ = 0.03). However, the univariate results only show a significant effect for “The process of COVID-19 testing works well in my class/classes” (F3, 1061 = 4.05, *p* < 0.01, Eta^2^ = 0.02) as well as for “I feel a heavy burden from regular COVID-19 testing at my school” (F3, 1061 = 9.74, *p* < 0.01, Eta^2^ = 0.03). For teachers in special schools (M = 3.26, SD = 0.87), the COVID-19 testing process was less satisfactory compared with elementary (M = 3.50, SD = 0.67; *p* < 0.01) and compulsory secondary school teachers (M = 3.59, SD = 0.62; *p* < 0.01). The difference between special school and academic secondary school teachers (M = 3.47, SD = 0.72) was not significant. Elementary school teachers (M = 2.71; SD = 0.96) experienced higher levels of additional stress due to the COVID-19 tests compared with compulsory secondary school teachers (M = 2.33; SD = 1.00, *p* < 0.01) and special education teachers (M = 2.42; SD = 0.99; *p* < 0.05). Compulsory secondary school teachers felt less burden than academic secondary school teachers (M = 2.63; SD = 1.04; *p* < 0.05) from regular COVID-19 testing. For “My students feel very burdened by regular COVID-19 testing at school”, no significant influence of school type was found (F3, 1061 = 2.14, *p* = 0.03, Eta^2^ = 0.01).

The Pearson correlation analysis indicated that more teaching experience is positively linked with a well-working testing process (r = 0.18, *p* < 0.01). However, the correlation is very small. No correlation was found between teaching experience and perceived level of burden (teachers themselves: r = 0.02, n.s.; students: r = −0.04, n.s.). Further, teachers’ job satisfaction was higher if COVID-19 testing worked out well (r = 0.18, *p* < 0.01) and if teachers perceived less burden on themselves (r = −0.26, *p* < 0.01) and on their students (r = −0.17, *p* < 0.01). Again, however, the correlation coefficients indicate that the strength of the effects is rather low.

## 4. Study 2

### 4.1. Participants

Five teachers participated in the current interview study. Participants were not selected according to a theoretical or criteria-based sampling method. Rather, in the course of the large-scale distribution of the online questionnaire, the teachers were asked to contact the researchers if they were interested in an interview-based study. This was because the researchers expected a state of emergency among educators due to COVID-19-related pedagogical challenges and therefore did not want to push teachers into participating. To provide a better overview of the teachers included in the subsequent data analysis, Table 2 lists their key characteristics.

### 4.2. Procedure

The qualitative method of guided interviews was chosen to collect the in-depth data. Considered a semi-structured approach to qualitative data collection [32], this is one of the basic methods of empirical social research [33]. Guided interviews can vary greatly in preparation and execution (e.g., the predetermined questions may be brief or detailed and interviewers may have more or less freedom in the wording or order of questions) [32]. However, all guided interviews are united by the principle of “as open as possible, as structured as necessary” ([33] p. 560, [34]), in that, on one hand, explicitly preformulated questions ensure a certain thematic focus, while, on the other hand, the interlocutor is given as much freedom as possible with regard to the course of the conversation.

The guide for the interviews conducted as part of this study was developed based on the theoretical foundation of the Organization for Economic Co-operation and Development (OECD)’s core dimension of teachers’ professional well-being [35], but also takes into account aspects relevant to collecting data on teachers’ experiences of the changes in the education sector brought about by COVID-19. Accordingly, respondents were asked about their subjectively perceived beliefs about their ability to cope with the challenging situation (i.e., cognitive well-being), their current emotions and moods (i.e., subjective well-being), their working conditions and associated stress levels (both physical well-being and psychological well-being), and the quality of communication and collaboration between teachers, between teachers and students, and between teachers and parents or guardians (i.e., social well-being) [35]. Finally, participating teachers were asked about the potential difficulties that the current school situation poses for vulnerable student groups (e.g., students with special education needs and socioeconomically disadvantaged students) and related trends of worsening educational inequity.

### 4.3. Data Analysis

For an in-depth analysis of the qualitative data, the documentary method according to Nohl (2009) [36] was used. This method originally goes back to the pioneering knowledge-sociological work of Mannheim (1980) [37] and has been substantially further developed by Bohnsack [38,39,40,41]. However, Nohl (2009) [36] was the first to apply the documentary method specifically to the analysis of interviews. Against the background of the basic idea of uncovering practical and everyday experiences of individuals or groups, the aim is to reconstruct tacit knowledge structures [36,41]. To achieve this objective, the first two steps of formulating and reflective interpretation are required. According to Nohl (2009) [36], this involves the selection of sections within the interviews based on the specific research interest. Subsequently, these text passages are transcribed, paraphrased, and briefly summarized as part of the formulating interpretation. This is followed by reflective interpretation, wherein the way in which certain topics are addressed by the interviewees (i.e., the orientation framework) is examined in more detail. To empirically capture the orientation framework, Nohl (2009) [36] next proposes a comparison of different interviews in which the same topic is treated differently. This is followed by the final step of type formation, which aims to identify orientation frameworks shared in multiple interviews. However, although this procedure implies an attempt at generalization, Bohnsack (1989) [38] points out that the formation of types is essentially based on the search for contrasts in commonalities, which leads to the creation of subtypes.

### 4.4. Results

#### 4.4.1. Teachers’ Evaluation of Different Measures to Curb the Incidence of COVID-19 Infections

During the data analysis, several themes were identified. However, in light of the chosen data analysis method, which requires the comparability of the data for subsequent typing, only the themes that appeared in all five interviews were examined in more detail. Those were: COVID-19 testing and wearing facemasks.

Teacher 1: COVID-19 testing is a challenge due to students’ lack of practice

When asked about key moments associated with the keywords “COVID-19”, “school”, and “emotions”, Teacher 1 answered: “I tend to think of funny things.”. This initial response suggests a subsequent positive narrative. However, Teacher 1 pointed out that the COVID-19 tests observed at baseline were very time-consuming, which she attributed to the lack of student practice: “It took us a long time to train the kids to the point where they were able to do the tests.”. Based on this description, it is clear that Teacher 1 attributed the reasons for the amount of time associated with the COVID-19 testing to external factors (e.g., students’ lack of practice). In addition, Teacher 1 placed at least some of the responsibility for the smooth administration of the COVID-19 tests on the students. This is expressed by the fact that while Teacher 1 referred to her efforts to get students used to regular testing (“It took us a lot of time to train the kids (…)”), she also referred to the need for students to be willing to make an effort (“(…) where they were able to take the tests.”). From further descriptions by Teacher 1, it appears that the tests took less time as the students became more proficient. This is also evident in the following phrase, in which Teacher 1 praised the currently prevailing testing procedure and compared it to military activities: “[N]ow it works like in the army (laughs).”. Finally, Teacher 1 put the previously expressed positive assessment of the current situation into perspective, again referencing the difficulties associated with the newly implemented COVID-19 testing due to problematic student actions. It is unclear at this point why Teacher 1 ended her statements with this somewhat negative review, although the COVID-19 tests—as previously described—worked smoothly during this time.

Teacher 2: COVID-19 testing as a bureaucratic burden

Teacher 2, who worked at an elementary school, described that at first it was difficult to perform the COVID-19 tests with young children. No further explanation was given as to why COVID-19 testing with students in elementary school was particularly challenging. It may be that some of the children lacked the fine motor skills necessary for testing (e.g., inserting the test sticks into the nose and opening the container with the test liquid). However, it could also be due to Teacher 2′s lack of competence in explaining to the children how to conduct the COVID-19 tests correctly. Next, Teacher 2 quickly put this first negative statement into perspective by saying that the testing procedure currently worked well and everyone had gotten used to it. Teacher 2 also stated that testing became a part of everyday life: “It’s normal, it’s part of everyday school life.”. Again, Teacher 2 pivoted from positive remarks and referred to the greatly increased amount of time required for testing concerning preparatory measures. Teacher 2 continued by referring to the testing as an additional bureaucratic burden. In addition, Teacher 2 identified the now mandatory PCR tests in particular as the trigger for the increased work: “It’s just extra bureaucratic work.”. These negative comments about the expenditure of time and bureaucratic burden seem surprising given the previous comment that the tests were going well. Perhaps Teacher 2 simply wanted to conclude his remarks by reiterating the workload associated with the COVID-19 testing.

Teacher 3: Facilitated COVID-19 testing due to students’ familiarity

Teacher 3 reported that the COVID-19 tests were initially feared by many, but that they eventually became a nonissue and worked smoothly. Teacher 3 did not specify at this point whom she meant by “many” and what they were specifically afraid of. Regarding the current COVID-19 testing, Teacher 3 explained: “I don’t find them so bad anymore; it goes very quickly. The kids already know the process and it works well.”. Teacher 3 did not directly cite the children’s proficiency as an argument for her subjectively perceived satisfaction with the way the COVID-19 tests were performed. However, it can be assumed that the mere mention of the students’ role in the testing process indicates that Teacher 3 wanted to express a connection between the students’ familiarity with the COVID-19 tests and the quality of their application. The participant then turned to the Ninja Passes (i.e., a document in which students’ COVID-19 tests were recorded and which was proof of permission to access all public areas), which Teacher 3 found extremely annoying at the beginning of their implementation: “The Ninja Passes annoyed me insanely at the beginning.”. This negative, past-tense narrative is complemented by the reasoning that Teacher 3 had repeatedly received emails from parents or guardians reporting missing stickers or lost Ninja Passes. This is followed by a more positive description of the current situation. Accordingly, Teacher 3 stated that he adapted to using the Ninja Pass check as an opportunity to connect and talk with students.

Teacher 4: Facilitated COVID-19 testing due to support from the school’s janitor

Teacher 4 commented on the organization of the implementation of the COVID-19 tests at her school site and reported that the preparation tasks were done by the school’s janitor: “The tests are well organized because we have a great school janitor who prepares everything for us.”. The term “we” implies a collectively shared assumption, meaning that this opinion was shared by other teachers working in the school. 

Teacher 5: Facilitated COVID-19 testing due to secretarial support

Teacher 5 first described her subjective response to the COVID-19 testing as follows: “Yes, the testing is fascinating. I would never have thought that this would all be my responsibility.”. This statement is contradictory in that the term “fascinating” can be considered synonymous with “captivating” or “intriguing” and therefore implies a subsequent positive narrative. However, the expressed overlap of COVID-19 testing with the teacher’s area of responsibility indicates a sense of being overwhelmed. Next, Teacher 5 described the current smooth implementation of the COVID-19 testing: “Now we take the boxes, we carry them into the classroom, and do the COVID-19 testing with the students. There are no problems, but it is easy for us because we have secretaries who prepare the boxes for us.”. This statement shows that Teacher 5 attributed the smooth implementation of the COVID-19 testing to external causes, namely to the secretaries’ support, and not to herself or the students. This is followed by a reference that puts the participant’s personal school situation—ostensibly perceived as privileged—into perspective. Accordingly, Teacher 5 stated that principals at other schools were tasked with administering the tests: “I personally know elementary school principals who do everything themselves, so all this administration, the lists, the sending, preparing the kits (…)”. It is questionable at this point why Teacher 5 brought up the example of the school principals and their situation. One possible interpretation would be that Teacher 5 wanted to express her knowledge of her own privileged situation (secretarial support). Furthermore, it is also conceivable that Teacher 5 intended to simply communicate her sympathy for those school principals. Next, Teacher 5 again referred to her personal situation and the time required for the tests: “[B]ecause we still spend 20 min of the lesson on COVID-19 testing, at least, but if I had to set everything up now, well, that’s, that’s insane.”. It is clear that Teacher 5 critiques the valuable lesson time lost due to the COVID-19 testing. However, the hypothetical assumption of having to do the preparatory and follow-up work in addition to the implementation of the COVID-19 tests itself was finally described by Teacher 5 as “insane”. This hyperbolic wording may serve to put her own situation into perspective once again. Finally, Teacher 5 praised the regularity of the PCR tests in the province of Upper Austria—the federal state in which the teacher worked—but she also emphasized the problem of the lack of support staff and the extreme difficulty that still accompanied teachers’ COVID-19 testing duties.

Teacher 1: Facemasks as a disruptor of teacher–student relationships

In her descriptions, Teacher 1 expressed that she felt better protected by wearing facemasks when vaccinations were not yet made available to teachers. Teacher 1 added, however, that the children noticed when she flinched at physical contact that was too close and quickly put on her facemask: “And the children notice that too, that you flinch and immediately put on the mask again.”. From this statement, it appears that Teacher 1 was afraid of close physical contact with students. Teacher 1 expressed that the students noticed her effort to distance herself, which, in turn, seemed to make her uncomfortable. One possible reason for this could be the close relationship between Teacher 1 and her students, which was disrupted by the hygiene measures implemented due to COVID-19.

Teacher 2: Facemasks as an imposition on young children and their teachers

In the interview, Teacher 2 briefly spoke several times about the difficulties associated with wearing facemasks for both students and teachers. Accordingly, Teacher 2 and his colleagues were very upset that children of elementary school age had to wear facemasks: “What bothers me and my colleagues the most at the moment is that the children have to wear a mask, that the teachers in elementary school have to wear a mask (…).”. At this point, Teacher 2 did not describe the specific problems associated with wearing facemasks in elementary schools. However, this becomes clearer in the next paragraph: “I can’t put a teacher in front of six-year-old children (…) who stands there with a mask and talks when they are just learning to speak and spell.”. Based on this quote, it is now obvious that Teacher 2 primarily wanted to point out linguistic difficulties. Thus, students who are just learning to speak, spell, write, and read would be denied observational learning. Finally, Teacher 2 also talked about the problems associated with wearing facemasks for teachers: “I wear the mask, the kids wear the mask. And I think everyone knows that it’s not so great to wear the mask when you have to talk a lot.”. Although Teacher 2 did not elaborate, it can be assumed that he is referring to physical complaints here. Finally, Teacher 2 revisited the linguistic problem already mentioned, but this time shed light on the educators’ perspective: “Conversely, I don’t understand the children so well, not all of them speak very loudly and very clearly, and then it becomes very problematic.”.

Teacher 3: Language and health problems caused by facemasks

Teacher 3 explained that she found the facemasks annoying. She justified this by referencing the language problems that affected both herself and the students: “What really annoys me are the masks (…) because the children can hardly understand me when I have the mask on. (…) I have to speak extremely loud.”. At this point, Teacher 3 took the students’ perspective and addressed the auditory difficulties associated with wearing facemasks. However, she quickly switched back to her own point of view at the next moment: “I have to speak extremely loud (…).”. Subsequently, Teacher 3 mentioned some health problems caused by wearing the facemask: “(…) it gives me a sore throat, a headache, it’s hot. It is very uncomfortable.”. In summary, Teacher 3 was primarily concerned with those language and health issues that directly affected herself. This is also evident from the fact that the students’ perspective was used to explain the teacher’s predicament. 

Teacher 4: Facemasks for students in fall 2021

Teacher 4 started by looking back to the summer of 2021, when she was “perfectly okay” with no facemasks in schools due to low infection rates, high vaccination rates, and the possibility of regular classroom ventilation. However, in the fall of 2021, Teacher 4 stated that she would have liked the students to wear facemasks: “Because now it’s just difficult with the ventilation, some get sick because it’s so cold in the school (…) and because the numbers have risen rapidly in the fall (…).”. Interestingly, Teacher 4 referred exclusively to students at this point, suggesting that she saw them as a potential threat to her own health, even though she did not mention this explicitly.

Teacher 5: Facemasks causing legal problems

Teacher 5 first referred to great difficulties associated with facemasks, which would have required actions from the Directorate of Education’s legal department: “(…) so there were a lot of problems that the Directorate of Education with the legal department helped us a lot with.”. Teacher 5 then more specifically explained that some complaints from parents or guardians were made through lawyers in letters to the principals: “I don’t know exactly, but there were letters written by some lawyers to the directors that this is forbidden and the Constitution does not allow it (…)”. With the initially vague formulation “I don’t know exactly”, Teacher 5 expressed her uncertainty, possibly because she wanted to emphasize her personal distance from these legal conflicts. Another reason for choosing this expression could be that Teacher 5 aimed to avoid more detailed questions from the interviewer. Next, Teacher 5 emphasized that the educators in her school were very accepting of facemasks. A short time later, however, Teacher 5 struck a different tone: “But lately I have noticed that it’s, uh, that it’s crazy now with the masks (…) you talk a lot in class and constantly inhale your exhaled air (…) I suddenly don’t know what I wanted to say. I walk to the window just for a moment, remove the mask (…) it’s okay again, but I notice that the mask is affecting me a lot at the moment.”. It is apparent that although Teacher 5 initially expressed her and her colleagues’ contentment with the facemasks, she then quickly turned to the personally perceived disadvantages.

#### 4.4.2. Typology of Teachers Based on Their Perceptions of Working Conditions during the COVID-19 Pandemic

Based on the preceding in-depth analysis of the participants’ statements, typologies were created considering teachers’ evaluation of two specific measures to curb the incidence of infections (i.e., COVID-19 testing and wearing facemasks) and the resulting consequences for their work conditions. 

##### Teacher Types regarding COVID-19 Testing

Type A: Rituals and routines as support for coping with new tasks due to responsibility expansion

Teachers 1, 2, and 3 addressed the associated time expenditure regarding the COVID-19 testing. Thus, the COVID-19-related hygiene measure implemented at schools required teachers to take the time to reliably conduct the tests with students in their classes. Since the COVID-19 tests had to take place in schools and always at the beginning of each school day, educators had to sacrifice some of their teaching time. This was seen as a major problem. Teacher 1 associated the issue of the time involved in COVID-19 testing with the students’ skills and proficiency. Accordingly, the reduction in time spent on COVID-19 testing and the associated additional workload depended largely on the extent to which students acquired the necessary skills to perform the tests, which required practice, explanations, and support. Through the introduction and implementation of standardized, routine sequences of actions, students’ testing performance was gradually perceived as satisfactory. In his comments on the time and workload expenditure of COVID-19 testing, Teacher 2 referred to the additional bureaucratic work. In line with Teacher 1, he emphasized the need for teachers and students to become accustomed to the new measures so that they could become part of everyday school life. Here, too, the ritualization of actions is stressed as an important component in mastering new tasks and the additional bureaucratic burden. In the interview with Teacher 3, a rather positive attitude toward COVID-19 testing emerges. According to Teacher 3, the more often students underwent the COVID-19 testing procedure, the more proficient they became, and the more they integrated it into their everyday school life. 

Type B: Smooth implementation of COVID-19 testing due to support staff

Both Teacher 4 and Teacher 5 reported positive experiences with the hygiene measure of regular COVID-19 testing introduced by education policymakers. Satisfaction with the COVID-19 tests was attributed to the fact that both teachers were supported by non-teaching staff who worked at their school. As for Teacher 4, the school’s janitor was responsible for administrative tasks, such as preparing the test kits. As for Teacher 5, the preparatory work was done by the school’s secretaries. Teachers 4 and 5 shared the (self-perceived) privileged position of having additional support on the COVID-19 tests, which presumably reduced their workload and time expenditure. 

##### Teacher Types regarding Facemasks

Type C: Facemasks protecting teachers

Teacher 1 and Teacher 3 both expressed their positive attitude toward wearing facemasks in schools. However, on closer inspection, the two teachers discussed this issue in different ways.

Subtype C.1: Teachers’ facemasks as a protective measure for teachers

In her statement about wearing facemasks in schools, Teacher 1 expressed a feeling of safety. Facemasks were perceived as a protective measure in times when vaccinations were not available to teachers. Therefore, facemasks were a temporary measure that provided a certain level of security for teachers. 

Subtype C.2: Students’ facemasks as a protective measure for teachers

With respect to Teacher 3, a positive attitude is reflected in a review of fall 2021. Accordingly, Teacher 3 stated that she would rather have students wear facemasks due to several framework conditions, among others, to protect her own health and to lower her risk of becoming infected herself. 

Type D: Facemasks as a source of problems 

Several teachers (2, 3, and 5) expressed their negative attitudes toward wearing facemasks in schools. 

Subtype D.1: Facemasks as a source of problems for teachers

Teacher 3 and Teacher 5 both expressed their concerns about facemasks with reference to issues that directly affected themselves.

Subtype D.1.1: Facemasks as a source of language and health problems for teachers

In her statement, Teacher 3 strongly focused on language and health problems associated with wearing facemasks in class. Teachers’ obligation to speak loudly in order for students to follow the lesson resulted in physical issues that were perceived as uncomfortable.

Subtype D.1.2: Facemasks as a source of cognitive problems for teachers

Teacher 5 explained that wearing a facemask in class caused cognitive problems. Inhaling her exhaled air through the facemask was perceived as a major problem and affected the teacher’s ability to stay focused in class.

Subtype D.2: Facemasks as a source of problems for students

This specific subtype could only be identified in Teacher 2, as he was the only one who discussed in detail the student-related issues related to wearing facemasks in schools. Forcing students who were just learning to speak, spell, write, and read to wear a facemasks was an unconscionable act and made observational learning impossible.

## 5. Discussion

Since the beginning of the global COVID-19 pandemic, schools have not solely been a place of education and socialization, but—like any other place that accommodates and fosters social interaction—a potential place for the spread of COVID-19 infections. Therefore, hygienic measures, such as wearing facemasks and regularly testing for the COVID-19 virus, were installed at the government level, and implementation was made mandatory for school staff. Within the current paper, in-depth insights into teachers’ perceptions about these measures are provided by using quantitative as well as qualitative methodological approaches.

According to the quantitative results, it became obvious that the implementation of hygienic measures and regulations to prevent COVID-19 infections was not working well in only a small number of classes. More teaching experience was associated with a more positive testing process. However, due to the small effect size, this result is of limited practical significance and application. Furthermore, special education teachers felt that administering the tests was more difficult compared with regular education teachers at both at the elementary and secondary school levels. This could be explained by the fact that special schools were not given special consideration by education policymakers in the development of protective measures. However, special schools are very diverse, and teachers are particularly challenged to address students’ situations. For example, some learners might be unable to complete the COVID-19 test independently or might have difficulty doing so due to physical or cognitive impairments. With this in mind, strategic plans for the implementation of COVID-19-related hygiene measures in special schools would have been appropriate and necessary.

Around half of the sample felt high levels of burden due to the testing procedure implemented in schools. However, teachers perceived lower levels of burden for students deriving from the regulations and hygienic measures in the context of education and school. One possible explanation might be that teachers perceived the testing procedure mainly as an occupational burden encompassing increased time expenditure as well as task shifting and enlargement, which was confirmed in the course of the qualitative results. Therefore, testing takes away important instructional time.

As mentioned in the literature review, the organization and implementation of COVID-19 testing procedures hold a high degree of potential for additional workloads and time expenditures [25]. In accordance with these assumptions, the analysis in the course of the present study reveals similar findings. Regarding teachers’ perception of the testing procedure in schools, it is noted as a pivot for their further perception of hygienic measures whether and to what extent teachers considered the organization and implementation of the testing as their task or whether and to what extent this task was attributed to them [24].

Referring to Unger et al. (2021) [23] stating that COVID-19 testing in schools should be feasible and efficient, the participating teachers’ perspectives changed from rather negative connotations to more positive attitudes as soon as all participants (especially teachers and students) gained sufficient competence in implementation through repetition and further established habit. This means that when testing was conducted under the teacher’s responsibility, the process was perceived as a challenge and an additional burden until it became a ritualized and routine procedure for both students and the teacher. These results emphasize the need for an institutional adaption phase allowing teachers and students to become accustomed to the new measures so they can become part of everyday school life. In this context, the ritualization of actions is highlighted by teachers as an important component in mastering new tasks. A settling-in period regarding new tasks seems to be a necessary as well as feasible demand on the school’s internal level as well as at the institutional level, provided that school operations can be run on a regular basis. In this way, the co-construction and co-creation of the “new normal” could be established in a joint process encompassing the perspectives, feedback, and ideas of the diverse stakeholders involved [42,43,44]. However, in the context of crisis-ridden upheavals, such as the COVID-19 pandemic, unpredictable changes and the resulting need for action make it difficult to introduce new regulations successfully. Due to the possibly unpredictable communication at the decision-making level with stakeholders in practice (school management staff, teachers, etc. (Woltran et al., submitted)) and the resulting sudden changes in the framework conditions of the education field and schools, there has been a lack of time resources for the prepared and reflective implementation of new regulations.

Regarding teachers’ different approaches to facemasks, the positive driver for wearing them or wanting others to wear them was a self-protective concern. With regard to the positive connotations of wearing a mask, the interviews did not reveal any motives resulting from altruism or solidarity with other persons present in the school building, but only self-serving strategies. It is interesting to note that regarding health-related reasons for wearing facemasks, teachers predominantly referred to their own health being protected by the regulation, especially against the background that previous studies have increasingly pointed out that the wearing of masks in schools as a hygiene measure has negative effects on students’ development [11,12,13,14,15]. Therefore, the finding of the current study does not support the results of previous studies reporting the importance of the social context and interactional altruism, meaning that people would wear facemasks to protect not only themselves but also others [45,46].

A possible explanation for the limitation to teachers’ self-protection reasons for wearing a mask (both one’s wearing and expecting others to wear a mask) is provided by the literature on motives for health behaviors showing that “anxiety encourages individuals to seek safety-promoting behaviors to protect themselves” ([47] p. 3; [48]), but can promote altruism when the outcome of a specific self-protecting behavior has a beneficial impact on future interactions with individuals profiting from these actions [49].

In line with this are the theoretical background results of the empirical study by Liekefett and Becker (2021) [50] showing that teachers’ self-protection motives during the pandemic are mostly based on coping with increased anxiety and stress [44,51,52,53], whereas group-protection motives are indicative of a group’s collective goal or identification with others [50]. In line with this theoretical background, teachers may have perceived themselves as one of the few occupational groups required to follow the state’s regulations regarding the organization of work without any independent, autonomous opportunities for co-determination. In the occupational field of schooling, for example, it was not possible to individually design home-office arrangements or flexible working regulations, as teachers and other educational staff had to follow governmental guidelines [54]. Within the narrative of the so-called system maintainers, teachers may not have identified with the basic needs and fears that the general population faced during the pandemic. Rather, they felt that their fears and anxieties were heightened by the systemically regulated work requirements and, therefore, they saw more of a need for self-protection. The newly constructed narrative of the system maintainers (in German: “Systemerhalter:innen” [55]) describes occupational groups that had to continue their regular work activities with few restrictions or adaptions to cover basic societal needs during the COVID-19 pandemic. In the case of school staff, the education system was maintained. Other studies refer to teachers as “frontline workers” during the COVID-19 pandemic when discussing the professional requirement of unquestioned presence in the work context when it was mandated by policymakers, resulting in heightened emotional and psychological challenges [51,54].

With regard to the negative aspects of wearing a mask during lessons, two of the participating teachers reported from a self-centered perspective, and only one teacher reported disadvantages for students regarding their opportunities for language learning and acquisition. Contrary to expectations, no statements made by the teachers directly referred to the effects of facemasks on the relationship between teachers and students. Against the background of the results of previous empirical studies [17,18,19], which found interference in social interaction to be one of the main negative consequences of mask-wearing, the lack of thematization by the participating teachers is surprising.

## 6. Conclusions 

Due to COVID-19 and the need to maintain schools as safe places, education policy in Austria has placed a strong focus on infection control processes. Against this backdrop, school staff had no choice but to comply with all requests from policy education stakeholders. As outlined in the current study, a large number of teachers experienced particular difficulties in implementing hygiene measures (i.e., regular COVID-19 testing and wearing facemasks) in their classrooms. This is not surprising given the additional administrative burden often associated with interventions to address COVID-19 infection rates in schools. However, it must be taken into account that wearing facemasks and performing COVID-19 tests were not entirely new for school staff. Accordingly, the wearing of facemasks in schools had already become mandatory for a certain period in the 2020–2021 school year. In addition, COVID-19 testing was conducted regularly in the months leading up to the 2021 summer holidays.

In summary, education policy continued to increase the testing frequency in the 2021–2022 school year but failed to take into account the additional workload it placed on teachers. In this context, it can be hypothesized that there is still a need for administrative staff in all types of schools, which is also in line with the findings of the National Education Report [56]. Accordingly, school principals have already indicated in an earlier survey in 2018 and 2019 that there is a need for personnel to undertake administrative tasks, especially in elementary and compulsory secondary schools. Accordingly, the results of the present study, in conjunction with the findings of previous surveys, lead to the conclusion that education policy in Austria should finally invest more financial resources in the staffing of schools. This is the only way to maintain the quality of the teaching profession and relieve the burden on teachers in the long term. Furthermore, concerning future pandemics and crises, it would be important to determine, within the framework of further large-scale research projects, which factors positively influence the implementation of hygiene measures and thus the perception by school staff. In this context, it would also be necessary to investigate the differences in teachers’ satisfaction with hygiene measures between different types of schools, as well as regional differences, to identify possible needs based on representative samples.

## Figures and Tables

**Table 1:** Descriptive Statistics.

Item	Strongly Disagree	Partly Disagree	Partly Agree	Strongly Agree	M (SD)
“The process of COVID-19 testing works well in my class/classes.”	2.1%	5.6%	33.4%	58.9%	3.49 (0.7)
“I feel a heavy burden from regular COVID-19 testing at my school.”	17.8%	31.3%	30.7%	20.2%	2.53 (1.01)
“My students feel very burdened by regular COVID-19 testing at school.”	19%	45.1%	28.3%	7.6%	2.25 (0.85)

**Table 2:** Characteristics of the Interviewed Teachers.

	Gender	School Type	Years of Teaching Experience
Teacher 1	Female	Public secondary school	35
Teacher 2	Male	Public elementary school	6
Teacher 3	Female	Private inclusive elementary and secondary school	5
Teacher 4	Female	Public secondary school	5
Teacher 5	Female	Public secondary school	24

## Data Availability

The data presented in this study are available on request from the corresponding author. The data are not publicly available due to sensitive information from which conclusions about the individual participants could be drawn.
